# Estimation of the economic impact of a bluetongue serotype 4 outbreak in Tunisia

**DOI:** 10.3389/fvets.2024.1310202

**Published:** 2024-02-29

**Authors:** Ameni Ben Salem, Elhem Ben Aicha, Sana Kalthoum, Anissa Dhaouadi, Haikel Hajlaoui, Bassem Bel Haj Mohamed, Imed Ben Slimen, Wiem Khalfaoui, Raja Gharbi, Kaouther Guesmi, Mehdi Ben Ali, Naouel Fatnassi, Chedia Seghaier, Thameur Ben Hassine, Mohamed Gharbi

**Affiliations:** ^1^Centre National de Veille Zoosanitaire, Tunis, Tunisia; ^2^Comméssariat au developpement agricole de Nabeul (CRDA), Direction générale des services vétérinaires (DGSV), Tunis, Tunisia; ^3^Laboratory of parasitology, Univ. Manouba, Ecole Nationale de Médecine Vétérinaire de Sidi Thabet. 2020, Sidi Thabet, Tunisia

**Keywords:** bluetongue, economic impact, outbreak, losses, Tunisia, sheep, goat, cattle

## Abstract

**Introduction:**

Since 1999, Tunisia has experienced multiple occurrences of Bluetongue (BT) outbreaks, leading to numerous reported cases of infection and mortality in flocks. The re-emergence of the disease in 2020 caused substantial economic losses in cattle, attributed to the incursion of serotype BTV-4.

**Methods:**

To evaluate the economic impact of the recent BT episode, we conducted a retrospective study on outbreaks that occurred in Tunisia between August and November 2020, focusing on the impact at the owner’s level and its effects on both small ruminants and cattle. A total of 234 ruminant farms (sheep, cattle, and mixed) were randomly selected across Tunisian governorates and included in the study to estimate both the direct and indirect costs of these outbreaks.

**Results:**

Total costs were calculated as the sum of losses and expenditures resulting from the BT outbreaks. At the animal level, total losses were estimated to range between 116.280 and 207.086 TND for one infected ewe (€33.721 and 60.055). For one lactating cow, costs varied between 2,590.724 and 3,171.107 TND (€751.310 and 919.621). In cattle, exposure to BTV led to a daily unit milk yield decrease of 12.50 to 14.66 L over an average period of 5 months. Diseased sheep experienced weight loss ranging between 4 and 10 kg during the BT outbreaks. The total mean cost of the 2020 BT outbreak in Tunisian investigated farms was estimated at 1,935 million TND (million €561.15) (range: 1,489 and 2,474 million TND; 431.81 and million €717.46). The most influential costs of the total BT outbreaks were the decrease in milk yield, mortality, and veterinary treatment.

**Discussion:**

This study gives valuable insights on the economic impact of the incursion of a new serotype of BT in a naive population in Tunisia. Considering the substantial costs incurred, it is imperative that this disease receives increased attention from stakeholders, including animal owners, veterinary services, practitioners, and decision-makers.

## 1 Introduction

Bluetongue (BT) is a viral disease, primarily transmitted to ruminants by biting midges (*Culicoides* spp.). However, alternative modes of transmission, such as the oral route, have been identified (1). The causative agent is the Bluetongue virus (BTV), classified within the *Orbivirus* genus belonging to the *Reoviridae* family. The virus is present on all continents except Antarctica (2). Both domestic ruminants (sheep, cattle, buffalo, goats, and camels) and wild ones (sambar, white-tailed deer, blue bulls, lamas, antelopes, etc.) have demonstrated susceptibility to BTV (2). To date, BTV includes 28 serotypes worldwide; serotypes 1–24 are considered classical among the prevalent serotypes, while the others are categorized as atypical, exhibiting milder clinical signs that primarily affect small ruminants (3). The complex epidemiology of various strains and susceptible species contributes to a highly variable impact in different contexts. Economic losses due to BT consist of direct costs at the farmer level (production losses, mortality, morbidity, and reduced fertility rate) and indirect costs at the national level (control and surveillance costs, trade restrictions, vaccination, diagnosis, and vector monitoring) (2). Since the 1960s, the World Organization for Animal Health (WOAH) has listed this arbovirus as notifiable due to its major economic losses (4, 5).

Global losses caused by BT outbreaks were estimated at 3 billion US dollars. In the US livestock industry, losses attributable to trade restrictions and the diagnosis of BTV were estimated at 144 million per year (6).

During the BTV-8 epizootic episode in Europe between 2006 and 2008, BT caused significantly higher economic losses than any previous BTV serotype epizootic. For example, in the Netherlands, the overall economic losses of BTV-8 outbreaks (BT-2007) were valued at 157–164 million € (5). In Germany, the mean impact of the BTV-8 outbreak was estimated at 180 million € (range: 157 and 203 million €) (7). In France, during 2007, the milk yield loss per BT diseased cow ranged between 111 and 249 kg per standard lactation (8).

Bluetongue virus (BTV) has also been reported in North African countries, including Morocco, Algeria, and Tunisia (9). In Morocco, BTV-10, BTV-4, and BTV-6 were identified in 1956, 2004, and 2006, respectively. Subsequently, various outbreaks have been documented (10). In Algeria, outbreaks caused by BTV-2 were reported in 2000 (9), while BTV-1 was concurrently identified in 2006, 2008, 2009, and 2010 (11).

In Tunisia, BTV-2 was first described in 1999, resulting in a total of 14,775 clinical cases and 1,286 deaths in sheep. Since then, the disease has become enzootic (12). From 1999 to 2016, the control strategy relied on mass vaccinating sheep using a live vaccine. However, from 2017 onward, an attenuated vaccine has been used to vaccinate animals against three serotypes: BTV-1, BTV-2, and BTV-4 (12).

Nevertheless, the control measures implemented proved insufficient, leading to subsequent outbreaks in Tunisia involving different serotypes: 2006 (BTV-1), 2009 (BTV-4), 2016 (BTV-3) (13), and 2017 (BTV-Y) (14).

Recent studies have shown that several BTV serotypes circulated simultaneously in Tunisia. This epidemiological situation raised concerns about the effectiveness of vaccination. Conventional vaccines, such as live-attenuated and inactivated vaccines, do not guarantee a cross-protective immune response among serotypes. Additionally, there is a significant variation in the virulence and symptoms of serotypes in different ruminant species (2, 15). As a result, mass vaccination of sheep in Tunisia has ceased since 2017.

The emergence of BT in different regions of the Mediterranean Basin, including Tunisia, has been primarily linked to climate change (16). Indeed, in 2020, increased rainfall and favorable temperatures led to an expansion in the vector population in Tunisia. This resulted in the re-emergence of BTV-4 in sheep, goats, and especially in cattle, leading to significant economic losses reported by farmers.

Despite the numerous outbreaks since its emergence in 1999 and the BT spreading over large parts of Tunisia, the financial impact of outbreaks has not been evaluated. The present study aims to estimate the economic impact of the BT 2020 outbreaks at the farmer’s level among Tunisian cattle and small ruminant farmers. Assessing the economic impact of BT is crucial for understanding the potential consequences at national and farmer levels, enabling informed decision-making and effective mitigation strategies to safeguard agricultural sustainability and economic resilience.

## 2 Materials and methods

### 2.1 Data collection

A retrospective study was conducted to examine the economic impact of the BTV outbreaks that occurred between August and November 2020 in Tunisia, affecting both small ruminants and cattle. The study involved a randomly selected sample of sheep, cattle, and mixed farms located in various Tunisian governorates. To ensure robust analysis, only farms with adequate data were retained. Verbal consent was obtained from all participating animal owners.

Data were collected from the BT-affected farms using a structured questionnaire, initially tested on five animal owners, and then adjusted accordingly. Information collection relied on the recollection of animal owners and insights from expert opinions, including private and government veterinarians actively engaged in bluetongue outbreak monitoring and control. Given that many of the included animal owners lacked a formal data recording system, information collection was based on animal owners’ memories and expert opinions (public and private veterinarians actively engaged in BT surveillance and control).

The collected data consisted of three main sections:

- Information related to the farms: this section includes data on the location farms [Governorate, delegation (an administrative subdivision of the Governorate)] as well as the typology of the farm (e.g., small ruminant, cattle, and mixed), the number of animals on the farm, and the species of animals present.
- Information related to BT outbreaks: the number of infected animals, the number of animals that died as a result of the disease, the date the first signs of the disease appeared, and the duration (days) of the disease, the number of pregnant females affected, and the number of females that aborted as a result of the disease.
- Information related to the economic impact of BT: this section encompassed the costs of; treating the disease, the costs of using insecticide for treatment, the decrease in milk yield, the costs associated with live weight decrease, the costs of abortion, the costs of mortality, and any expenses related to purchasing replacement animals.

Data were entered into an Access® database, and statistical analysis was carried out using MedCalc® software for Windows, version 20.014 (MedCalc Software, Ostend, Belgium), and Excel software® for Windows®.

### 2.2 Sensitivity analysis

To address the imprecision in economic construction costs and the variability of others, we developed a stochastic spreadsheet model for economic analysis and sensitivity analysis using @Risk (Palisade Corporation) software for Excel® version 8.2. All uncertain data values were represented as distributions, and details are provided in Supplementary Table S1.

The model was run with 10,000 iterations to capture the range of potential outcomes, and a confidence level of 95% was considered.

Additionally, we ranked each cost item based on its contribution to the total costs. The results of the sensitivity analysis were evaluated through a tornado plot, providing a visual representation of how the mean fluctuates across the spectrum of each input variable.

### 2.3 Estimation of epidemiological indicators

Three epidemiological indicators were calculated for distinct farm groups—specifically small ruminant farms, cattle farms, and mixed farms—according to the following formulas:


$$\begin{array}{l} \mathrm{Morbidity rate}\;\left(\%\right)=100\times \mathrm{Number of diseased animals in}\;a\;\mathrm{farm}/\\ \mathrm{total number of animals} \end{array}$$

$$\begin{array}{l} \mathrm{Mortality rate}\;\left(\%\right)=100\times \mathrm{Number of dead animals in}\;a\;\mathrm{farm}/\\ \mathrm{total number of animals} \end{array}$$

$$\begin{array}{l} \mathrm{Case fatality rate}\;\left(\%\right)=100\times \mathrm{Number of dead animals in}\;a\;\mathrm{farm}/\\ \mathrm{number of diseased animals} \end{array}$$

### 2.4 Cost estimation

The evaluation of the impact of the BT focused exclusively on losses sustained at the farm level, encompassing only losses incurred by farmers. Laboratory costs were omitted from the evaluation as the Tunisian government covered the expenses related to labor, sampling materials, and laboratory procedures (including PCR costs). All financial values were expressed in Tunisian Dinars (TND) and subsequently converted into euros (€) using the exchange rate as of July 2023 (1 TND=€0.29), as reported by the Tunisian Central Bank in 2023 (17).

#### 2.4.1 Treatment costs (C_TRA_)

Total treatment costs are derived by aggregating the individual treatment expenses per farm, calculated as follows:


$$C_{\mathrm{TRA}}={\mathrm{VET}}_{\mathrm{private}}+C_{D}+C_{\mathrm{TRA}-\mathrm{farmers}}$$

Where,

C_TRA_: treatment costs
VET_private_: private field veterinarian fees
C_D_: drug costs
C_TRA-farmers_: costs of treatments applied by farmers

#### 2.4.2 Costs of insecticide treatments (C_INT_)

Farmers applied Deltamethrin and/or Cypermethrin to treat both animals and premises. The total cost of insecticide treatments (C_INT_) is equal to the sum of the estimated costs for treating animals and premises for each animal type, categorized by farm type (cattle, small ruminants, cattle in mixed farms, and small ruminants in mixed farms). It was calculated as follows:


$$C_{\mathrm{INT}}={\mathrm{Pr}}_{f}.\left(C_{\mathrm{INT}-\mathrm{animals}}+C_{\mathrm{INT}-\mathrm{premises}}\right)+C_{L}$$

Where,

Pr_f_: proportion of farmers who bought the insecticides
C_INT-animals_: cost of applying insecticides to the animals
C_INT-premises_: cost of animal premises’ treatment with insecticides
C_L_: Labor costs.

C_INT-animals_ relies on factors such as the unit price of 1 mL of insecticide (P_INT_), the volume of used insecticide per animal (V_A_), the number of animals treated (N_AT_), and the number of applications per animal (N_AA_), formulated as follows:


$$C_{\mathrm{INT}-\mathrm{animals}}=P_{\mathrm{INT}}.V_{A}.N_{\mathrm{AT}}.N_{\mathrm{AA}}$$

For C_INT-premises_, it included the unit price of 1 mL insecticide (P_INT_), the volume of insecticide needed to treat 1 m^2^ of premise (0.5 mL), the number of applications per premise (N_AP_), and the area of premises treated in m^2^ (P_area_):


$${\mathrm{INTC}}_{\mathrm{premises}}=P_{\mathrm{INT}}.0.5.N_{\mathrm{AP}}.P_{\mathrm{area}}$$

The corresponding labor costs were estimated by summing the hours of labor needed to apply insecticide to animals (HLa) and in animal premises (HLp), multiplied by the unit price of 1h of labor (PHL):


$$C_{L}=\left(H_{\mathrm{La}}+H_{\mathrm{Lp}}\right).P_{\mathrm{HL}}$$

Where,

H_La_: hours of labor needed to apply insecticide to animals
H_Lp_: hours of labor needed to apply insecticide in the animal premises
P_HL_: unit price of 1 h of labor

According to the farmer’s opinion, the average cost of 1 h of labor was 3.720 TND (€1.079). A single insecticide treatment for one cattle required between 2.30 and 3.52 min, as indicated by Gharbi et al. (18). Application of a single insecticide treatment for cattle required an estimated time ranging between 2.30 and 3.52 min, as indicated by Gharbi et al. (18). According to expert input, the same treatment for each small ruminant was approximated to take between 0.2 and 1 min. Additionally, the application of insecticide treatment to a 10-m^2^ area was estimated to necessitate between 2 and 3 min of labor.

#### 2.4.3 Production losses

##### 2.4.3.1 Estimation of milk yield decrease (C_MYD_)

The decrease in milk yield among female small ruminants was not calculated, as their milk is not commercialized on the majority of Tunisian farms.

Milk yield losses due to BTV in lactating cows (C_MYD_) were estimated as follows:


$$C_{\mathrm{MYD}}=\left(A_{\mathrm{DM}}{-}_{\mathrm{bBT}}-A_{\mathrm{DM}}{-}_{\mathrm{dBT}}\right).N_{\mathrm{days}}.P_{M}$$

Where,

A_DM_-_bBT_: average daily milk yield before the BT episode
A_DM_-_dBT_: average daily milk yield during the BT episode
N_days_: number of days with milk yield decrease
P_M_: market price of 1 L of milk sold by farmers in Tunisia during 2020. It has varied between 0.9 and 1.3 TND (€0.261 and 0.377), according to the governorates

##### 2.4.3.2 Estimation of live weight decrease (C_LWD_)

Losses resulting from the decrease in live weight were only considered for sheep. For cattle, our primary focus was on the milk yield decrease since the majority of surveyed animals were dairy. The price range of 1kg of sheep live weight was obtained from the Office de l’élevage et des pâturages (OEP) in 2020 (19). The mean losses due to the live weight decrease were estimated as follows:


$$C_{\mathrm{LWD}}=\left(A_{\mathrm{LW}}{-}_{\mathrm{ha}}-A_{\mathrm{LW}-\mathrm{ia}}\right).N_{\mathrm{days}}.P_{\mathrm{LW}}$$

Where,

A_LW_-_ha_: average live weight per healthy animal
A_LW-ia_: average live weight during the BT outbreak per infected animal
N_days_: number of days with live weight decrease
P_LW_: market price of 1 kg of live weight during 2020. It ranged between 10.4 and 11.3 TND (€3.016 and 3.277) (OEP, 2020)

##### 2.4.3.3 Abortion costs (C_ABO_)

We attributed all abortions during a BT outbreak to BTV infection if the disease occurred during pregnancy. We assumed that the cost of one abortion equaled the market price of a newborn animal of the same species. Total losses due to abortion were estimated as follows:


$$C_{\mathrm{ABO}}=N_{\mathrm{AF}}.P_{\mathrm{NA}}$$

Where,

N_AF_: number of aborted females
P_NA_: average market price of a newborn animal of the same species. The P_NA_ for a newborn lamb, goat, and calf were estimated at 110 (€31.9), 73 (€21.2), and 400 TND (€116), respectively

##### 2.4.3.4 Mortality costs (C_MOR_)

Mortality costs were estimated as follows:


$$C_{\mathrm{MOR}}=\mathrm{ND}.\mathrm{Mv}$$

These deaths were ranked by species as follows: deaths in calfs (ND_c_), cows (ND_cw_), lambs (ND_l_), sheep (ND_s_), goat kids (ND_gk_), and adult goats (ND_g_). The market value (Mv) of animals was estimated according to expert opinion (Table 1).

**Table 1:** Market value of different animal categories.

Animal categories	Range of market value of one animal in TND (€)	Mode of market value of one animal in TND (€)
Calves	[1,200-2,500] (348–725)	1,250 (362.5)
Cows	[950–5,500] (275.5–1,595)	2000 (580)
Lambs	[260–450] (75.4–130.5)	275 (79.75)
Ewes	[300–1,000] (87–290)	400 (116)
Goat kids	[200–350] (58–101.5)	250 (72.5)
Adult goats	[300–650] (87–188.5)	350 (101.5)

#### 2.4.4 Estimation of total costs

Total costs (C_TOT_) were calculated as the sum of losses and expenditures caused by the BT outbreak as follows:


$$C_{\mathrm{TOT}}=\sum \left(\begin{array}{l} C_{\mathrm{TRA}}+C_{\mathrm{INT}}+C_{\mathrm{MYD}}+C_{\mathrm{LWD}}\\ +C_{\mathrm{ABO}}+C_{\mathrm{MOR}}+P_{\mathrm{RA}} \end{array}\right)\;$$

Where,

C_TOT_: total costs
C_TRA_: treatment costs
C_INT_: insecticide treatment costs
C_MYD_: milk yield decrease costs
C_LWD_: live weight decrease costs
C_ABO_: abortion costs
C_MOR_: mortality costs
P_RA_: purchases of replacement animals

## 3 Results

### 3.1 Characterization of the surveyed farms

A total of 234 farmers were surveyed, overseeing a population of 40,395 ruminants. This comprised 38,274 small ruminants and 2,121 cattle (Table 2). BTV presence was confirmed through PCR analysis in over half of the farms (155/234; 66.2% **±** 0.96). For the remaining 79 farms, no laboratory tests were conducted as animals exhibited clear clinical signs of BT.

**Table 2:** Typology of investigated farms having declared BT outbreaks.

Farm type	Number of farms (%)	Number of cattle	Number of small ruminants	Total ruminants’ population	Range of age [months- years]
Small ruminant farms	126 (53.8)	0	28,142	28,142	[1 month to 10 years]
Cattle farms	12 (5.1)	299	0	299	[3 months to 8 years]
Mixed farms^*^	96 (41)	1,822	10,132	11,954	[1 month to 10 years]
Total	234	2,121	38,274	40,395	-

^*^Sheep, goats, and cattle.

The surveyed animals were raised on three types of farms: small ruminant farms (sheep and goat) (53.8%); cattle farms (5.1%); and mixed farms (sheep, goat, and cattle) (41%) (Table 2). The median ages of small ruminants and cattle were 4 years (range: 1 month–10 years) and 4.5 years (range: 3 months–8 years), respectively.

### 3.2 Epidemiological indicators

The overall morbidity within the studied ruminant population was estimated at 16.32% ± 0.002 (6,593/40,395). The mortality and case fatality rates were 3.28% ± 0.001 (1,326/40,395) and 20.11% ± 0.005 (1,326/6,593), respectively. Notably, the highest mortality rate was reported in small ruminants residing on mixed farms (*p* < 0.01). In comparison to the other farm types, the morbidity rate was significantly lower in cattle farms (*p* < 0.01) but came with the highest case fatality rate (*p* < 0.01) (Table 3).

**Table 3:** Morbidity, mortality, and lethality rates in investigated farms having declared BT outbreaks.

Rates	Morbidity rate (% ± SE)	Mortality rate (% ± SE)	Case fatality rate (% ± SE)
Small ruminant farms	4,196/28,142 (14.91 ± 0.002)	768/28,142 (2.73 ± 0.001)	768/4,196 (18.30 ± 0.006)
Cows in cattle farms	17/299 (5.69 ± 0.013)	5/299 (1.67 ± 0.007)	5/17 (29.41 ± 0.11)
Small ruminants in mixed farms	2,053/10,132 (20.26 ± 0.004)	419/10,132 (4.14 ± 0.002)	419/2,053 (20.41 ± 0.009)
Cows in mixed farms	327/1822 (17.95 ± 0.009)	134/1822 (7.35 ± 0.006)	134/327 (40,98 ± 0.27)
Total farms	6,593/40,395 (16.32 ± 0.002)	1,326/40,395 (3.28 ± 0.001)	1,326/6,593 (20.11 ± 0.005)

SE, Standard Error.

### 3.3 Cost estimation

#### 3.3.1 Treatment costs (C_TRA_) in farms with BT outbreaks

**Figure 1:**
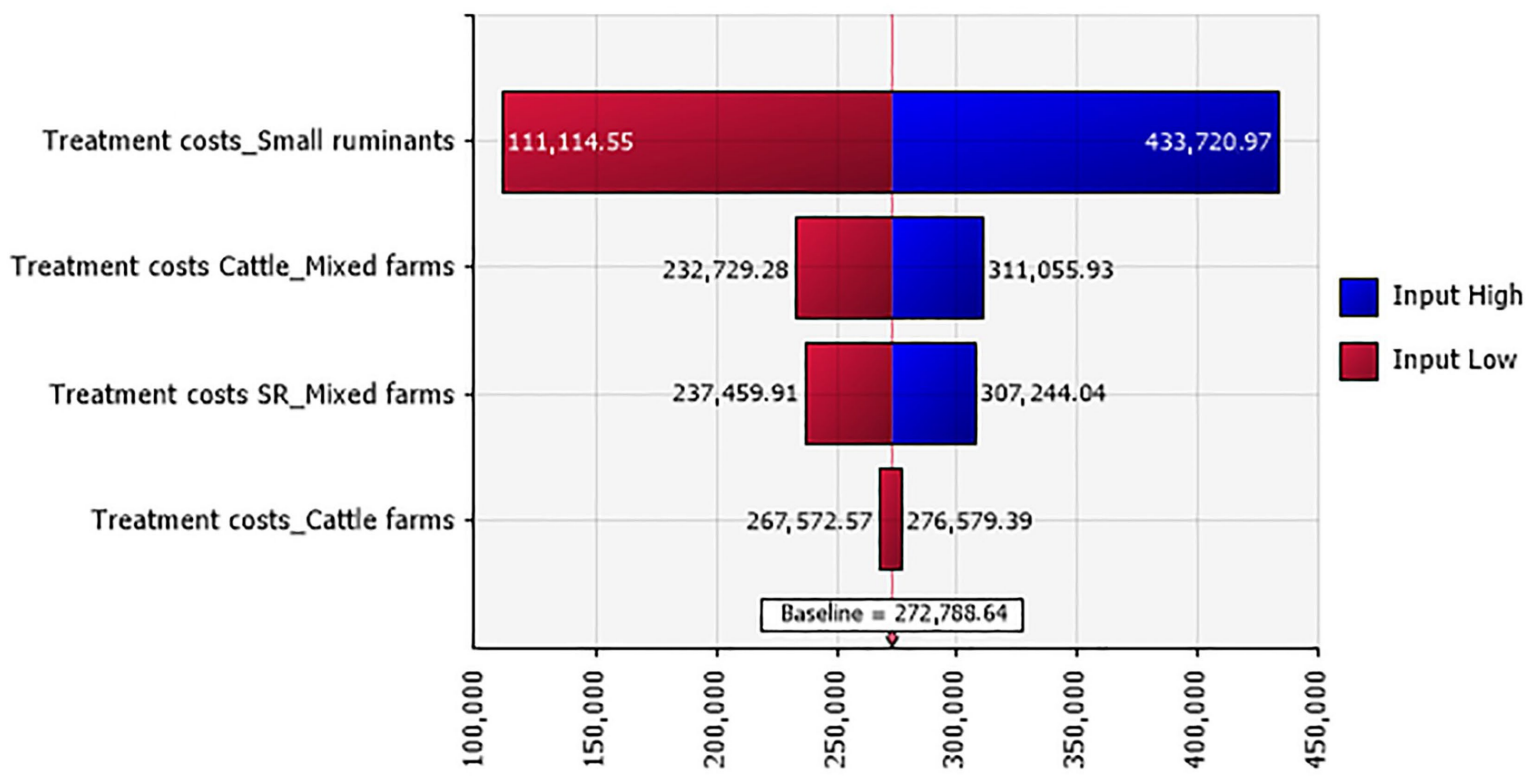
Tornado plot for the treatment costs (in TND) in Tunisian investigated farms according to their types.

A total of 4,891 ruminants were treated with antimicrobials, anti-inflammatory drugs, and antiseptics, uncurring an overall cost of 272,788.640 TND (€79,108.706). Treatment costs in small ruminant farms accounted for the highest proportion of the total treatment costs (Figure 1). In specific numbers, 220 cattle were treated, with a mean unit treatment cost varying between 211.191 and 231.142 TND (€61.245 and 67.031). Additionally, 4,671 clinical small ruminant cases received treatment, with a unit cost ranging between 45.953 and 56.643 TND (€13.326 and 16.426).

#### 3.3.2 Insecticide treatment costs (C_INT_) in farms with BT outbreaks

During the episode of BT in 2020, 76.92% (180/234) of the farmers treated their animals and/or barns with insecticides. The farmers individually purchased 90% of the total quantity of insecticide used, while the government covered the remaining 10%. The overall insecticide treatment costs ranged between 11,255 and 27,408 TND (€3,263.950 and 7,948.320). The cost of insecticide treatment for one small ruminant during the BT outbreak varied between 0.327 and 0.443 TND (€0.095 and 0.128). For cattle, this cost ranged between 0.991 and 1.143 TND (€0.287 and 0.331).

#### 3.3.3 Estimation of production losses due to the BT outbreak

##### 3.3.3.1 Estimation of milk yield decrease (C_MYD_) due to the BT outbreak

The daily milk yield decrease per diseased cow was estimated to range between 12.50 and 14.66 L (Table 4), corresponding to a market value between 13.75 and 16.13 TND (€3.988 and 4.678). The total cost of milk yield losses during the BT outbreak for the 217 cows varied between 192,000 and 865,000 TND (€55,680 and 250,850).

**Table 4:** Cost of milk yield decrease in cows due to BT outbreak in Tunisian investigated farms.

Item	Cattle farms	Cattle in mixed farms
Mean unit price of 1 L of milk	1.1	1.1
Mean milk yield decrease/infected cow/day [L]	14.66	12.50
Mean period of milk yield decrease [days]	152	152
Mean milk yield decrease /infected cow/day [TND] (€)	16.13 (4.678)	13.75 (3.988)
Milk yield decrease/infected cow/BT outbreak [TND] (€)	2,438.822 (707.258)	2,078.542 (602.777)
Number of infected lactating cows	9	208
Total costs of milk yield decrease [TND] (€)	21,949.40 (6,365.210)	432,336.667 (125,377.633)

##### 3.3.3.2 Estimation of live weight decrease (C_LWD_) due to the BT outbreak

Several symptoms contribute to weight loss in diseased sheep (Table 5). Total losses attributed to live weight loss varied between 13,983 and 28,981 TND (€4,055.070 and 8,404.490).

**Table 5:** Losses of liveweight decrease in sheep due to BT outbeak in Tunisian investigated farms.

Item	Sheep in small ruminant farms	Sheep in mixed farms
Range of the price of 1 kg ovine liveweight [TND] (€)	[10.4–11.3] (3.224–3.503)	[10.4–11.3] (3.224–3.503)
Weight loss/diseased sheep/BT outbreak [kg]	(1, 7, 9, 16–21)	(1, 7, 9, 16–21)
Losses due to weight decrease/diseased sheep [TND] (€)	75.950 (22.026)	75.950 (22.026)
Number of diseased sheep with liveweight diseased	216	67
Total costs of live weight decrease [TND] (€)	16,405.200 (4,757.508)	5,088.650 (1,475.709)

##### 3.3.3.3 Losses due to abortion (C_ABO_) in BT diseased females

**Figure 2:**
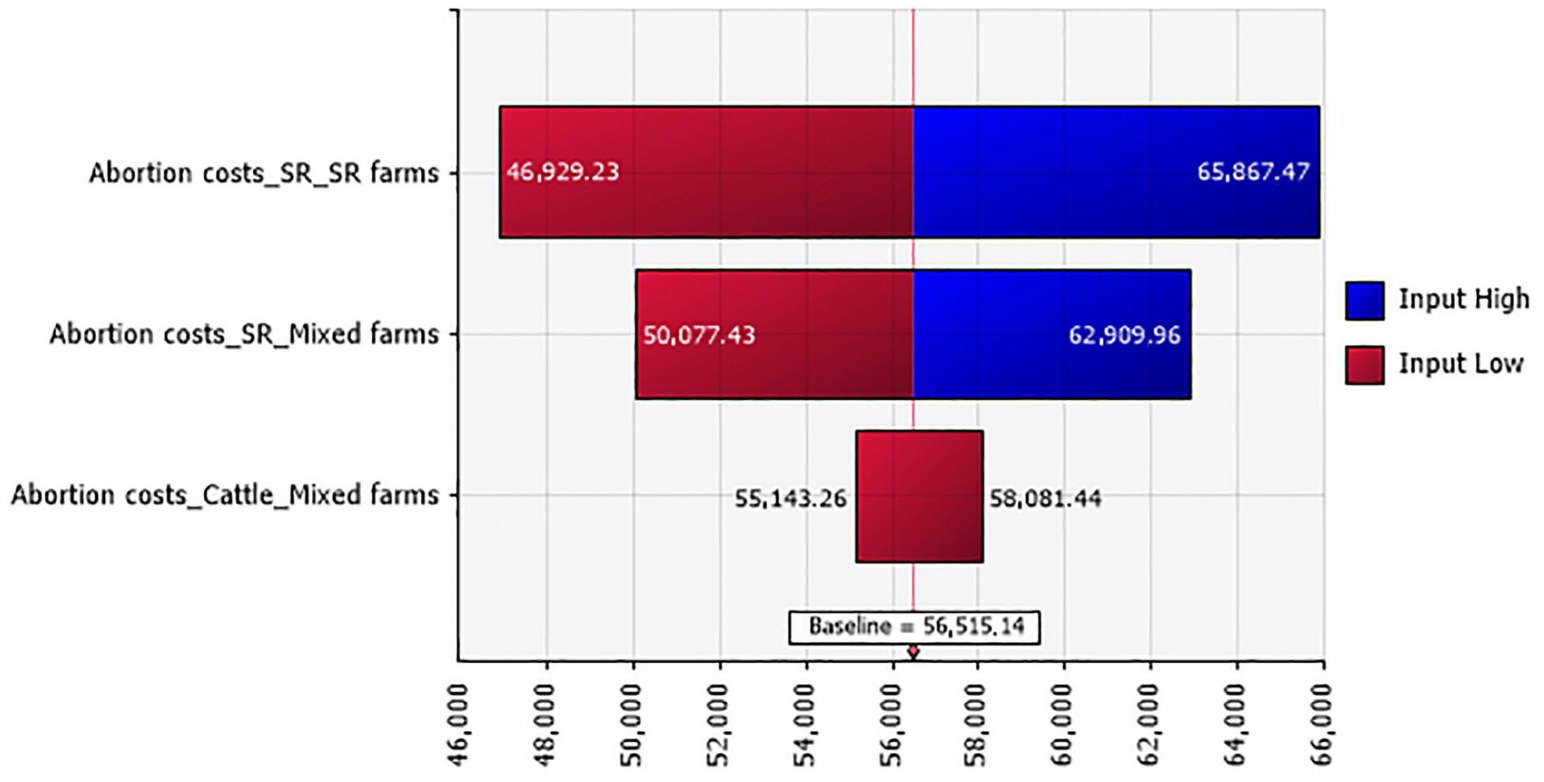
Tornado plot for the abortion costs (in TND) in Tunisian investigated farms according to their types.

The overall abortion rates for small ruminants and cattle in BT diseased animals were estimated at 16.81% ± 0.007 (444/2,642) and 22.62% ± 0.046 (19/84), respectively. Total losses due to abortions were evaluated to be between 42,644 and 70,527 TND (€12,366.760 and 20,452.830), and abortion costs for small ruminants contributed with the highest percentage to the total abortion losses (Figure 2).

##### 3.3.3.4 Mortality costs (C_MOR_)

**Figure 3:**
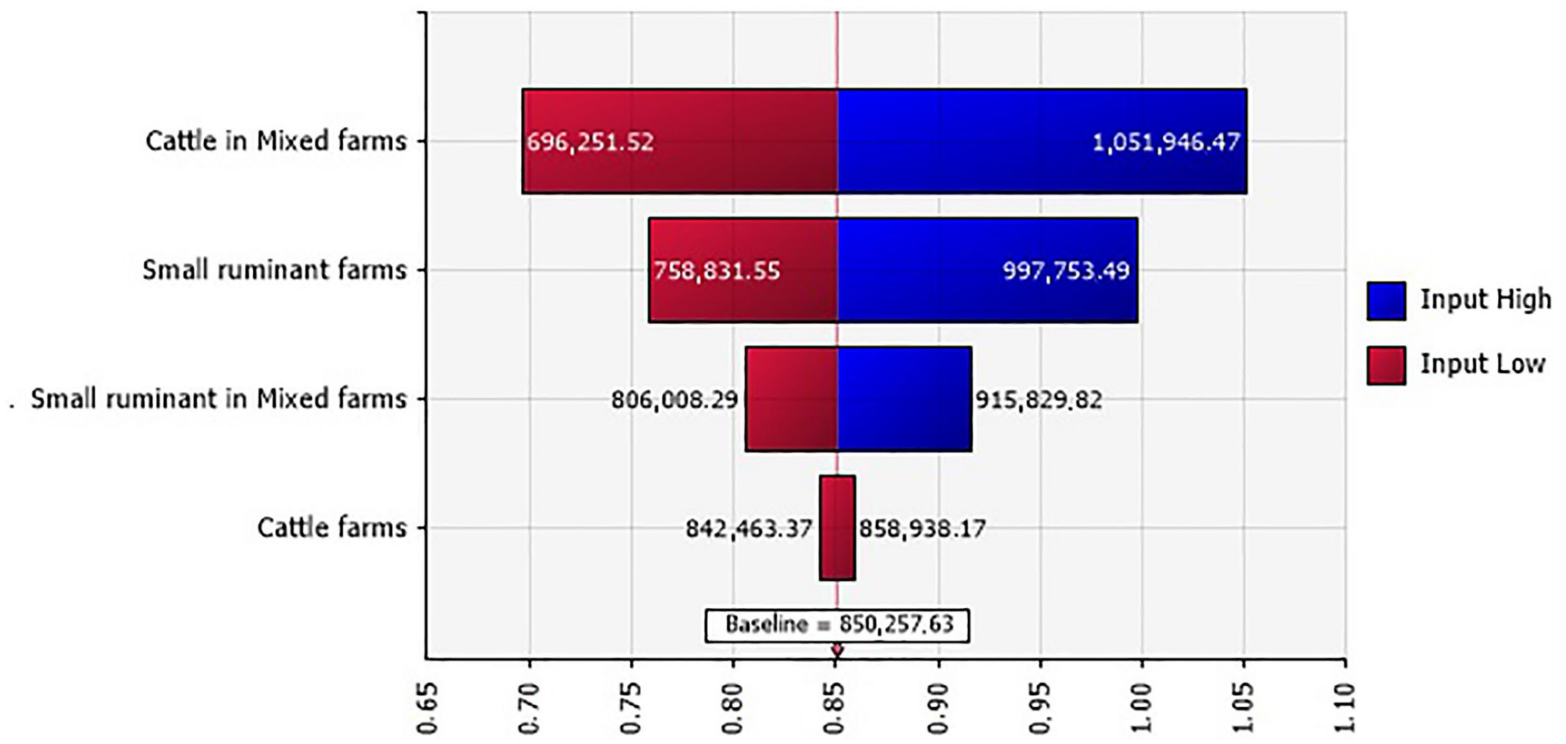
Tornado plot for the mortality costs (in million TND) in Tunisian investigated farms.

Following the BT outbreak, a total of 1,179 small ruminants and 139 cattle succumbed to the disease. The overall losses due to mortality varied between 654,000 and 1,084,000 TND (€189,660 and 314,360). When considering farm types, mortality costs for cattle in mixed farms held the highest proportion of the total mortality cost (Figure 3).

#### 3.3.4 Purchase of replacement animal costs (C_PRA_)

Due to the death of animals during the BT outbreak, expenses incurred for the acquisition of new ewes, cows, and goats amounted to an estimated total of 262,425 TND (€76,103.250).

### 3.4 Economic indicators and total costs

Total losses associated to the BT outbreak were approximated to range between 116.280 and 207.086 TND for one infected ewe (€33.721 and 60.055). Costs for one lactating cow varied between 2,590.724 and 3,171.107 TND (€751.310 and 919.621) (Table 6).

**Table 6:** Economic losses due to the BT outbreak per infected animal estimated by sensitivity analysis.

Animal category	Item	Minimum and maximum cost/animal in TND (€)	Mean cost/animal in TND (€)	Percentage
Lambs	Treatments	[45.953–56.643] (13.326–16.426)	51.298 (14.876)	40.19
	Insecticides	[0.327–0.443] (0.095–0.128)	0.385 (0.112)	0.3
	Liveweight decrease	[43.4–108.5] (12.586–31.465)	75.95 (22.026)	59.51
	Total	[89.680–165.586] (26.007–48.020)	30.4 (8.816)	
Ewes	Treatments	[45.953–56.643] (13.326–16.426)	51.298 (14.876)	31.73
	Insecticides	[0.327–0.443] (0.095–0.128)	0.385 (0.112)	0.24
	Abortions	[70–150] (20.300–43.500)	110 (31.900)	68.03
	Total	[116.280–207.086] (33.721–60.055)	161.683 (46.888)	
Calves	Treatments	[211.191–231.142] (61.245–67.031)	221.17 (64.139)	99.52
	Insecticides	[0.991–1.143] (0.287–0.331)	1.067 (0.309)	0.48
	Total	[212.182–232.285] (61.533–67.363)	222.23 (64.447)	
Cows	Treatments	[211.191–231.142] (61.245–67.031)	221.17 (64.139)	7.68
	Insecticides	[0.991–1.143] (0.287–0.331)	1.067 (0.309)	0.04
	Milk yield decrease	[2,078.542 - 2,438.822] (602.777–707.258)	2,258.682 (655.018)	78.40
	Abortions	[300–500] (87.000145.000)	400 (116.000)	13.88
	Total	[2,590.724-3,171.107] (751.310–919.621)	2,880.919 (835.447)	

**Figure 4:**
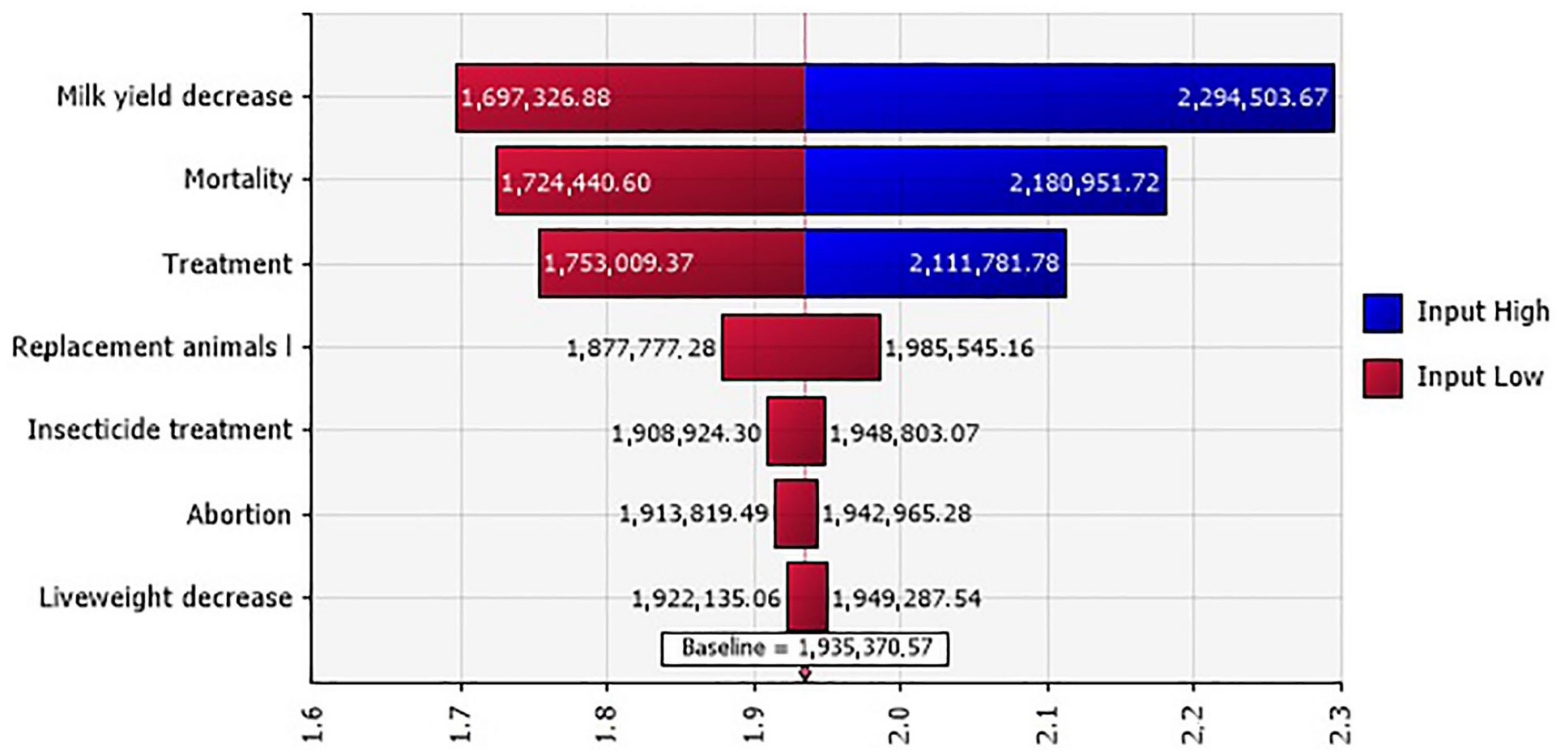
Tornado plot for the total BT outbreak costs (in million TND) in Tunisian investigated farms.

The total mean costs of the 2020 BT outbreak in the investigated farms in Tunisia were estimated at 1,935 million TND (million €561.15) within a range of 1,489–2,474 million TND (€431.81–717.46 million). A sensitivity analysis highlighted that the most influential costs for the total BT outbreak include milk yield decrease, mortality, and veterinary treatment (Figure 4).

## 4 Discussion

While BT is endemic in Tunisia, with a few cases reported annually, the noteworthy event in 2020 involved the introduction of serotype 4 in cattle. The registration of around a 100 outbreaks during that year prompted substantial inquiries into the economic implications of the disease.

This study aims to evaluate the economic impact of BT in affected farms in Tunisia. To the best of our knowledge, it is the first study to quantify the economic losses attributable to BT in Tunisia at the farm level. Recognizing that the losses incurred in 2020 primarily affected farmers, the study exclusively focuses on the farm level to comprehensively examine the economic impact of BT. The considered losses included: treatment costs (C_TRA_), insecticide treatment costs (C_INT_), milk yield decrease costs (C_MYD_), live weight decrease costs (C_LWD_), abortion costs (C_ABO_), mortality costs (C_MOR_), and purchases of replacement animals (P_RA_). However, some losses were not included due to a lack of data, such as costs induced by reproductive disorders (increased calving intervals, fertility decrease in rams, lower birth weights, and stillbirths) and additional labor charges (extra time spent feeding diseased animals, checking their health status, and treating wounds) (5). The majority of the current analyses were carried out by categorizing the animals into four distinct groups: small ruminant farms (encompassing sheep and goats), small ruminants within mixed farms, cattle farms, and cattle within mixed farms. It is important to note that this classification allows for a more nuanced examination of the economic impact, providing insights into the specific challenges faced by each group.

In small ruminant farms, the mortality rate was estimated at 2.73%. Notably, a comparable rate was reported during the BTV-4 outbreak in Spain (2.2%), according to Gómez-Guillamón et al. (20).

The highest mortality rate (7.35%) and case fatality rate (40.98%) were observed in cows living in mixed farms. This phenomenon could be explained by the sustained viral circulation among animals of different species, including cattle, sheep, and goats, particularly non-vaccinated naive cattle livestock. Additionally, this could be attributed to the preference of *Culicoides* biting midges for cattle over sheep, as highlighted by Bartsch et al. (22).

The case fatality rate in small ruminants, standing at 18.30%, was similar to that observed in southeastern European countries in 2014, resulting from the same serotype of BTV (ranging from 8.5 to 37.0%) (23). Indeed, the BTV-4 strain is most likely a reassortant variant derived from BTV-1, BTV-2, and BTV-4 isolates, circulating in the west Mediterranean and North Africa, as highlighted by Katsoulos et al. (24). However, the recorded case fatality rate in our study exceeded that documented in Tunisia (BTV-2) during the outbreak of 1999 [9%; (1,286/14,775)] (12). This difference could be attributed to the variations in the specific BT serotypes involved.

The mean treatment cost per cattle varied between 211.191 and 231.142 TND (€61.245 and 67.031). For small ruminant clinical cases, the unit cost varied between 45.953 and 56.643 TND (€13.326 and 16.426). These figures exceeded veterinary treatment costs in Germany during the BTV-8 outbreak (€10 per sheep; €26 per dairy cattle), as reported by Gethmann et al. (7). High costs of veterinary drugs (antimicrobials and anti-inflammatory), mostly imported overseas, may explain this high cost.

Notably, no preventive vector control measures were implemented before the BTV-4 outbreak in Tunisia. Seventy-seven farmers treated their animals and/or the premises with insecticides only during the clinical episode. Given that BT is transmitted by biting midges (*Culicoides* spp.), early treatment of animals with insecticides is crucial, as emphasized by Adam et al. (25), who reported a sevenfold decrease in BT seropositivity with cattle treated with insecticides (25).

The daily milk yield decrease per diseased cow was estimated between 12.50 and 14.66 L, corresponding to a market value between 13.75 and 16.13 TND (€3.988 and 4.678). It is important to note that agalactia was detected in 12% of the infected cows in our study. This decrease surpassed that observed in infected cows (5.4 kg/day) in the Netherlands during the BTV-8 outbreaks between 2006 and 2007 (5).

Even if the clinical episode does not exceed 10 days (range: 7–10 days), the BTV-4 infection induced a milk yield decrease over an average period of 5 months. This pattern was observed in France during the 2007 BT outbreak, where a significant decrease in milk yield persisted for over 6 months (8). The extended duration can be attributed to the prolonged healing time of lesions affecting teats, mouth, and feet (8).

Farmers reported a live weight decrease of 4–10 kg per infected sheep. In the Netherlands, the weight loss from July 2007 to July 2008 was estimated at 8% per animal (5).

Abortion is one of the main symptoms of BT, estimated at 16.81% in the current study. A similar abortion rate of 15.7% was reported in Belgium during the lambing period of 2008 (26). However, in São Paulo state, Brazil, abortion emerged as a predominant reproductive disorder, affecting over half of the ewes (53%; 74/139) (27).

The abortion rate in cattle (22.62%) exceeded that reported in the Nièvre district, France (16%) during the 2008–2009 BTV-8 outbreak (28). This disparity might be attributed to the potential overestimation of abortions linked to BT in our study, as the causative factor for the abortions was not confirmed through laboratory tests.

Total losses caused by the BT outbreak for one infected ewe were estimated to range between 116.280 and 207.086 TND (€33.721 and 60.055). These costs encompassed expenses related to abortion, treatment, and insecticide treatment. This result was lower than the reported mean direct cost per infected ewe in Germany (€74), which took into account reduced revenues for lamb sales (€59) and expenditure for veterinary treatment, especially after abortions (€10), were mainly considered in the total cost per animal (7). The total cost per sheep was even lower during the BT outbreak in India in 2019 and 2020 (€4.374) (29).

For one lactating cow, costs varied between 2,590.724 and 3,171.107 TND (€751.310 and 919.621), with the primary cost attributed to the milk yield decrease (78.4%). In Germany, the total direct loss from each BT-infected animal ranged between €119 and 136. Losses per animal included expenditure on restocking of elite animals (€99), treatment (€26), milk yield decrease (€24), and calf sales (€18) (7).

The sensitivity analysis highlighted that milk yield decrease, mortality, and veterinary treatment were the three most influential costs for the total BT outbreak costs. These findings were closely aligned to those reported in the Netherlands during the 2007 BT outbreak, where production losses and veterinary treatment fees represented 92% of the total cost (5). It is essential to note that our study only considered costs at the farmer level, while cost-effectiveness analyses in Switzerland (21) and Germany (7) included government expenses (control and surveillance costs, trade restrictions, vaccination, diagnosis, and vector monitoring). Preventive and control measures have a higher financial impact than the production losses caused by the disease itself (6).

While our study provides valuable insights into the economic impact of the BTV-4 outbreak in Tunisia in 2020, it is important to acknowledge several limitations. The reliance on data obtained from farmers introduces the possibility of recall bias, and variations in data accuracy and availability among participants may affect the overall robustness of our findings. The absence of laboratory confirmation for certain outcomes, such as abortion rates, and the exclusion of broader government costs in the analysis represent additional constraints. Future research should address these limitations to enhance the comprehensiveness and applicability of economic evaluations related to bluetongue outbreaks.

## 5 Conclusion

This study provides a comprehensive assessment of the economic impact of the BTV-4 outbreak in Tunisia, considering various cost factors and their implications on different livestock categories. The nuanced analysis, encompassing treatment costs, insecticide treatment costs, milk yield decrease costs, live weight decrease costs, abortion costs, mortality costs, and purchases of replacement animals, provides a holistic understanding of the economic losses caused by BT outbreaks at the farm level. The sensitivity analysis reaffirms the pivotal role of milk yield decrease, mortality, and veterinary treatment as key contributors to the total economic impact. This study not only contributes novel insights to the understanding of BT’s economic consequences but also establishes a foundation for future investigations in this field.

The findings underscore the importance of preventive measures, early detection indicators and contribute to informed decision-making and effective strategies for safeguarding livestock and farmers.

## Data availability statement

The original contributions presented in the study are included in the article/Supplementary material; further inquiries can be directed to the corresponding author.

## Ethics statement

Ethics approval was not required for this study because there was no manipulation with the animals by the investigators. Only information about the economic impact (veterinary treatment, mortality, abortion, milk production…) was collected during the period of the study. Verbal eclaired consents were obtained from all animal owners that participated in the study.

## Author contributions

AB: Conceptualization, Formal analysis, Investigation, Software, Writing – original draft, Writing – review & editing. EB: Conceptualization, Investigation, Writing – original draft. SK: Conceptualization, Formal analysis, Writing – original draft. AD: Investigation, Software, Writing – original draft. HH: Investigation, Writing – original draft. BB: Investigation, Writing – original draft. IB: Investigation, Writing – original draft. WK: Investigation, Writing – original draft. RG: Investigation, Writing – original draft. KG: Investigation, Writing – original draft. MB: Investigation, Writing – original draft. NF: Software, Writing – original draft. CS: Conceptualization, Writing – original draft. TB: Software, Writing – original draft. MG: Writing – review & editing.

## References

[1] Islam S Rahman MK Abedin J Zamil S Sayeed MA Rahman MZ. Serological evidence of bluetongue virus and associated factors in small ruminants of Bangladesh. Prev Vet Med. (2023) 211:105821. doi: 10.1016/j.prevetmed.2022.105821, PMID: 36584566

[2] Saminathan M Singh KP Khorajiya JH Dinesh M Vineetha S Maity M. An updated review on bluetongue virus: epidemiology, pathobiology, and advances in diagnosis and control with special reference to India. Vet Q. (2020) 40:258–321. doi: 10.1080/01652176.2020.1831708, PMID: 33003985 PMC7655031

[3] Ries C Domes U Janowetz B Böttcher J Burkhardt K Miller T. Isolation and cultivation of a new isolate of BTV-25 and presumptive evidence for a potential persistent infection in healthy goats. Viruses. (2020) 12:983. doi: 10.3390/v12090983, PMID: 32899808 PMC7552037

[4] Pascual-Linaza AV Martínez-López B Pfeiffer DU Moreno JC Sanz C Sánchez-Vizcaíno JM. Evaluation of the spatial and temporal distribution of and risk factors for bluetongue serotype 1 epidemics in sheep Extremadura (Spain), 2007-2011. Prev Vet Med. (2014) 116:279–95. doi: 10.1016/j.prevetmed.2014.05.009, PMID: 24929438

[5] Velthuis AG Saatkamp HW Mourits MC de Koeijer AA Elbers AR. Financial consequences of the Dutch bluetongue serotype 8 epidemics of 2006 and 2007. Prev Vet Med. (2010) 93:294–304. doi: 10.1016/j.prevetmed.2009.11.007, PMID: 19962204

[6] Rushton J Lyons N. Economic impact of bluetongue: a review of the effects on production. Vet Ital. (2015) 51:401–6. doi: 10.12834/VetIt.646.3183.1, PMID: 26741252

[7] Gethmann J Probst C Conraths FJ. Economic impact of a bluetongue serotype 8 epidemic in Germany. Front Vet Sci. (2020) 7:65. doi: 10.3389/fvets.2020.00065, PMID: 32118078 PMC7034324

[8] Nusinovici S Souty C Seegers H Beaudeau F Fourichon C. Decrease in milk yield associated with exposure to bluetongue virus serotype 8 in cattle herds. J Dairy Sci. (2013) 96:877–88. doi: 10.3168/jds.2012-5800, PMID: 23261379

[9] Hammami S. North Africa: a regional overview of bluetongue virus, vectors, surveillance and unique features. Vet Ital. (2004) 40:43–6. PMID: 20419633

[10] Daif S El Berbri I Lhor Y Fassi FO. Serological and molecular prevalence study of bluetongue virus in small domestic ruminants in Morocco. Sci Rep. (2022) 12:19448. doi: 10.1038/s41598-022-24067-y, PMID: 36376352 PMC9663439

[11] Madani H Casal J Alba A Allepuz A Cêtre-Sossah C Hafsi L. Animal diseases caused by orbiviruses. Algeria Emerg Infect Dis. (2011) 17:2325–7. doi: 10.3201/eid1712.110928, PMID: 22172371 PMC3311186

[12] Kalthoum S Sghaier S Ben Hassine T Teodori L Spedicato M Guesmi K. Risk-based serological survey of bluetongue and the first evidence of bluetongue virus serotype 26 circulation in Tunisia. Vet. Med Sci. (2022) 8:1671–82. doi: 10.1002/vms3.818, PMID: 35510402 PMC9297743

[13] Sghaier S Hammami S Goffredo M Hammami M Portanti O Lorusso A. New species of the genus Culicoides (Diptera Ceratopogonidae) for Tunisia, with detection of bluetongue viruses in vectors. Vet Ital. (2017) 53:357–66. doi: 10.12834/VetIt.986.5216.2, PMID: 29307131

[14] Lorusso A Sghaier S Di Domenico M Barbria ME Zaccaria G Megdich A. Analysis of bluetongue serotype 3 spread in Tunisia and discovery of a novel strain related to the bluetongue virus isolated from a commercial sheep pox vaccine. Infect Genet Evol. (2018) 59:63–71. doi: 10.1016/j.meegid.2018.01.025 29386141

[15] Jiménez-Cabello L Utrilla-Trigo S Calvo-Pinilla E Moreno S Nogales A Ortego J. Viral Vector Vaccines against Bluetongue Virus. Microorganisms. (2020) 9:42. doi: 10.3390/microorganisms9010042, PMID: 33375723 PMC7823852

[16] Jacquot M Nomikou K Palmarini M Mertens P Biek R. Bluetongue virus spread in Europe is a consequence of climatic, landscape and vertebrate host factors as revealed by phylogeographic inference. Proc Biol Sci. (2017) 284:20170919. doi: 10.1098/rspb.2017.0919, PMID: 29021180 PMC5647287

[17] Tunisian Central Bank (2023). Nation’s accounts. Available at: https://www.bct.gov.tn/bct/siteprod/index.jsp (Accessed July 2023).

[18] Gharbi M Touay A Khayeche M Laarif J Jedidi M Sassi L. Ranking control options for tropical theileriosis in at-risk dairy cattle in Tunisia, using benefit-cost analysis. Rev Sci Tech. (2011) 30:763–78. doi: 10.20506/rst.30.3.2074, PMID: 22435189

[19] Office de l’élevage et du pâturage (2020). Indicateurs clés des filières agricoles en Tunisie 2020.Observatoir national de l’agriculture. 42.

[20] Gómez-Guillamón F Caballero-Gómez J Agüero M Camacho-Sillero L Risalde MA Zorrilla I. Re-emergence of bluetongue virus serotype 4 in Iberian ibex (*Capra pyrenaica*) and sympatric livestock in Spain, 2018-2019. Transbound Emerg Dis. (2021) 68:458–66. doi: 10.1111/tbed.13696, PMID: 32573968

[21] Häsler B Howe KS Di Labio E Schwermer H Stärk KD. Economic evaluation of the surveillance and intervention programme for bluetongue virus serotype 8 in Switzerland. Prev Vet Med. (2012) 103:93–111. doi: 10.1016/j.prevetmed.2011.09.013, PMID: 22018548

[22] Bartsch S Bauer B Wiemann A Clausen PH Steuber S. Feeding patterns of biting midges of the Culicoides obsoletus and Culicoides pulicaris groups on selected farms in Brandenburg. Germ Parasitol Res. (2009) 105:373–80. doi: 10.1007/s00436-009-1408-y, PMID: 19308450

[23] Schulz C Sailleau C Bréard E Flannery J Viarouge C Zientara S. Experimental infection of sheep, goats and cattle with a bluetongue virus serotype 4 field strain from Bulgaria, 2014. Transbound Emerg Dis. (2018) 65:e243–50. doi: 10.1111/tbed.12746, PMID: 29119690

[24] Katsoulos PD Giadinis ND Chaintoutis SC Dovas CI Kiossis E Tsousis G. Epidemiological characteristics and clinicopathological features of bluetongue in sheep and cattle, during the 2014 BTV serotype 4 incursion in Greece. Trop Anim Health Prod. (2016) 48:469–77. doi: 10.1007/s11250-015-0974-5, PMID: 26768893

[25] Adam IA Abdalla MA Mohamed ME Aradaib IE. Prevalence of bluetongue virus infection and associated risk factors among cattle in North Kordufan state, Western Sudan. BMC Vet Res. (2014) 10:94. doi: 10.1186/1746-6148-10-94, PMID: 24762138 PMC4022533

[26] Saegerman C Bolkaerts B Baricalla C Raes M Wiggers L de Leeuw I. The impact of naturally-occurring, trans-placental bluetongue virus serotype-8 infection on reproductive performance in sheep. Vet J. (2011) 187:72–80. doi: 10.1016/j.tvjl.2009.11.012, PMID: 20061168

[27] Rizzo H Balaro MFA Matos ACD Lobato ZIP Gregory L. Is bluetongue virus a risk factor for reproductive failure in tropical hair sheep in Brazil? Acta Sci Vet. (2021) 49:1812. doi: 10.22456/1679-9216.112591

[28] Zanella G Durand B Sellal E Breard E Sailleau C Zientara S. Bluetongue virus serotype 8: abortion and transplacental transmission in cattle in the Burgundy region, France, 2008-2009. Theriogenology. (2012) 77:65–72. doi: 10.1016/j.theriogenology.2011.07.015, PMID: 21872306

[29] Senthil Kumar S Serma Saravana Pandian A. Estimation of economic losses due to bluetongue disease in sheep farms. J Entomol Zool Stud. (2020) 8:853–6.

