# Supplementary material for: Estimation of the economic impact of a bluetongue serotype 4 outbreak in Tunisia
Source: Front Vet Sci. 2024 Feb 29;11:1310202. doi: 10.3389/fvets.2024.1310202 (PMC10937385; doi:10.3389/fvets.2024.1310202)
Supplement: Supplementary file 1 [file Data_Sheet_1.docx]

**Table S1. Distribution and inputs for each parameter involved in the BT costs**

| **Parameter** | **Distribution form** | **Under parameters** | **Inputs** | **Distribution form** |
| --- | --- | --- | --- | --- |
| **Treatment costs** | BetaGeneral | Treatment costs in small ruminant farms | Treatment costs / small ruminant | Uniform |
|  |  | Treatment costs in cattle farms | Treatment costs / cattle | Uniform |
|  |  | Treatment costs for small ruminants in mixed farms | Treatment costs / small ruminant | Uniform |
|  |  | Treatment costs for cattle in mixed farms | Treatment costs / cattle | Uniform |
| **Insecticide treatment costs** | InvGauss | Cost of applying insecticides to a small ruminant | Total volume of used insecticide per a small ruminant | Uniform |
|  |  |  | Number of applications per a small ruminant | Pert |
|  |  | Cost of small ruminant premises’ treatment with insecticides | Number of applications per a premise | Pert |
|  |  |  | Area covered by a small ruminant in m^2^ | Uniform |
|  |  | Labour costs in small ruminant farms | Number of applications per a small ruminant | Pert |
|  |  |  | Area covered by a small ruminant in m^2^ | Uniform |
|  |  |  | Area of premises treated in m^2^ | Uniform |
|  |  |  | Number of applications per a premise | Pert |
|  |  | Cost of applying insecticides to a cattle | Total volume of used insecticide per a cattle | Uniform |
|  |  |  | Number of applications per a cattle | Pert |
|  |  | Cost of cattle premises’ treatment with insecticides | Number of applications per premise | Pert |
|  |  |  | Area covered by a cattle in m^2^ | Uniform |
|  |  | Labour costs in cattle farms | Number of applications per a cattle | Pert |
|  |  |  | Area covered by a cattle in m^2^ | Uniform |
|  |  |  | Area of premises treated in m^2^ | Uniform |
|  |  |  | Number of applications per premise | Pert |
| **Milk yield decrease costs** | Gamma | Milk yield decrease costs | Market price of 1 L of milk (TND) | Uniform |
|  |  |  | Average daily milk yield decrease per infected cow | Pert |
|  |  |  | Number of days with milk yield decrease | Pert |
| **Live weight decrease costs** | BetaGeneral | Live weight decrease costs | Average live weight decrease per infected sheep | Uniform |
|  |  |  | Number of days with live weight decrease | Pert |
| **Abortion costs** | Pert | Cost of cow abortions | Average market price of a newborn calf (TND) | Uniform |
|  |  | Cost of sheep abortions | Average market price of a newborn lamb (TND) | Uniform |
|  |  | Cost of goat abortions | Average market price of a newborn goat (TND) | Uniform |
| **Mortality costs** | BetaGeneral | Mortality cost in cattle | Market value of a cow | Pert |
|  |  |  | Market value of a calf | Pert |
|  |  | Mortality cost in sheep | Market value of a sheep | Pert |
|  |  |  | Market value of a lamb | Pert |
|  |  | Mortality cost in goats | Market value of adult goat | Pert |
|  |  |  | Market value of a goat kid | Pert |
| **Purchases of replacement animals** | BetaGeneral | Purchases of replacement cows | Price of a new cow | Uniform |
|  |  | Purchases of replacement ewes | Price of a new ewe | Uniform |
|  |  | Purchases of replacement goats | Price of a new goat | Uniform |
